# A case report of an intermediate phenotype between congenital myasthenic syndrome and D-2- and L-2-hydroxyglutaric aciduria due to novel *SLC25A1* variants

**DOI:** 10.1186/s12883-020-01854-6

**Published:** 2020-07-13

**Authors:** Wenhui Li, Min Zhang, Linmei Zhang, Yiyun Shi, Lei Zhao, Bingbing Wu, Xihua Li, Shuizhen Zhou

**Affiliations:** 1 Department of Neurology, Children’s Hospital of Fudan University, 399 Wanyuan Road, Shanghai, 201102 China; 2 Center for Molecular Medicine, Children’s Hospital of Fudan University, Shanghai, China; 3 Key Laboratory of Birth Defects, Pediatrics Research Institute, Children’s Hospital of Fudan University, Shanghai, China

**Keywords:** Congenital myasthenic syndrome, *SLC25A1*, D-2- and L-2-hydroxyglutaric aciduria, Phenotype

## Abstract

**Background:**

Variants in the *SLC25A1* gene are associated with a severe neurometabolic disease, D-2- and L-2-hydroxyglutaric aciduria (D/L-2-HGA). A report in 2014 presented the first account of congenital myasthenic syndrome (CMS) with mild intellectual disability (ID) caused by *SLC25A1*. To date, only two missense variants in *SLC25A1* have been linked to CMS.

**Case presentations:**

A Chinese boy presented fatigable muscular weakness, myasthenic crisis, epilepsy and developmental delay along with mild elevation of urinary 2-ketoglutarate (2-KG) and lactic acid levels. He showed a partial response to pyridostigmine. Genetic analysis using trio whole-exome sequencing (WES), Sanger sequencing, and cosegregation analyses revealed two novel pathogenic variants of *SLC25A1* (c.628C > T, p.R210X; c.145G > A, p.V49M).

**Conclusions:**

We report a boy who carries novel compound heterozygous variants of *SLC25A1* and presents a phenotype intermediate between CMS and D/L-2-HGA. This case expands the range of known phenotypes and genotypes associated with *SLC25A1*.

## Background

Congenital myasthenic syndrome (CMS) is a heterogeneous group of disorders caused by mutations that lead to impairment of neuromuscular transmission, mainly by affecting the components of the neuromuscular junction (NMJ). These disorders are usually manifested as early-onset fatigable ocular, bulbar and limb muscle weakness. However, increasingly recognized, the recently discovered CMS genes result in some complex manifestations that make the diagnosis more difficult.

*SLC25A1* encodes the mitochondrial citrate carrier SLC25A1 (CIC) [1]. Variants in the *SLC25A1* gene are associated with a severe neurometabolic disease, D-2- and L-2-hydroxyglutaric aciduria (D/L-2-HGA), which is characterized clinically by severe developmental delay, hypotonia, and seizures [2–4]. In 2014, two British siblings presenting with mild intellectual disability (ID) had been reported firstly to be complicated by CMS due to a homozygous (c.740G > A; p. R247Q) missense variant in *SLC25A1* [5]. Another four patients with the same missense variant in *SLC25A1* were identified as having mild CMS with ID in 2019 [6]. In addition to the recurrent reported pathogenic variant (c.740G > A; p.R247Q), the other missense variant (c.205G > T; p. D69Y) was recently confirmed in three siblings with CMS [7].

To date, only two missense variants in *SLC25A1* have been associated with CMS. We report a patient with an intermediate phenotype between CMS and D/L-2-HGA due to two novel compound heterozygous variants. We review the clinical and genetic features of CMS and further delineate the phenotype and genotype of this syndrome.

## Case presentation

The patient was diagnosed and fully investigated through clinical information, an electrophysiological examination and muscle biopsy at the Children’s Hospital of Fudan University, Shanghai, China. The patient is an 8-year-old male, the second child of healthy nonconsanguineous Chinese parents who also have a healthy 15-year-old daughter. The boy was born at term in an uneventful delivery, with a birth weight of 3.9 kg. At birth, he was noticed to have laryngeal stridor and a quiet cry.

At midnight one night when the boy was 9 months old, he was found with blue lips and an unresponsive stare. After approximately 10 min, he became conscious and felt tired. This event was not initially considered to be epileptic, as the child’s electroencephalograms (EEGs) were normal. However, two similar episodes manifesting as focal impaired awareness seizures were noted at 1 year old and 6 years old. There were some focal epileptic discharges on the boy’s EEG after the second seizure. Because his seizures were not very frequent, we did not prescribe any antiepileptic drugs to him.

On follow-up, developmental delay was noted. The patient could control his head at 4.5 months, sit without support at 8 months, and walk independently at 2 years. He could speak at 16 months, but his speech was inarticulate. The Gesell Developmental Schedules were applied at 3 years and 2 months old, and his scores were 66, 54, 51, and 72 in the social, adaptive, language, and motor areas, respectively. At present, he has learning difficulties and is unable to receive mainstream schooling.

After 18 months of age, he was noticed to move his head rather than his eyes to look upward. He developed intermittent bilateral ptosis and occasional strabismus at 23 months; both conditions were aggravated by exercise and improved with rest. After 2 years, his parents found that he had an unsteady gait and fatigable weakness of the limbs and face.

The patient had two myasthenic crises during follow-up. At 4 years and 8 months, he was admitted to the emergency department for sudden dyspnoea and exacerbation of limb weakness following acute bronchitis. He was placed on mechanical ventilation and an antibiotic regimen for several days. In the winter of 2018, he was diagnosed with pneumonia that presented as a fever and cough. He did not improve after taking oral antibiotics for 6 days. He abruptly presented dyspnoea and dysphonia on admission. Eventually, he recovered through ventilator-assisted breathing. His lactate levels were only mildly elevated in both crises.

Examination revealed some facial weakness with mild to moderate bilateral ptosis and ophthalmoplegia. The patient also had fatigable limb weakness. He was hypotonic, and his reflexes were normal.

Basic laboratory parameters were normal, including blood count, hepatic and renal function, creatine kinase, amino acid profile and acylcarnitine profile assays. Two urinary organic acid analyses at 2 and 8 years old all revealed a mild elevation of 2-ketoglutarate (2-KG) and lactic acid. Four measurements of blood lactate from 2 to 8 years were slightly elevated, ranging from 2.5 to 4.0 mmol/L (reference range 0.7–2.1). The patient was negative for acetylcholine receptor (AChR) antibodies.

Echocardiograms, electrocardiograms, and brain MRI were normal. Muscle biopsy suggested atrophy of type IIB muscle fibres with normal cytochrome c oxidase (COX) and succinate dehydrogenase (SDH) staining, and the electron microscopy results were normal, with no mitochondrial changes (Supplementary data). Repetitive nerve stimulation of the axillary nerve and median nerve at 3 Hz showed 25.1 and 14.3% decreases in compound muscle action potentials (CMAPs), respectively.

The patient was given pyridostigmine at 3 years of age, although he had some other features unrelated to CMS. His parents reported that pyridostigmine was partially effective, but he did not improve with a further trial of salbutamol. The daily dosage of pyridostigmine was increased from the initial 45 mg to the current 180 mg. Initially, there was no improvement with 45 mg per day of pyridostigmine, and the dosage was gradually increased to 90 mg per day after 8 months. The parents said the boy’s ptosis was alleviated and his overall physical activity was increased. After 2 weeks of observation, salbutamol was added at a dosage of 2 mg per day. In August 2015, pyridostigmine and salbutamol were tapered off to determine the effect. During the three-week observation period, the patient showed more severe ptosis, dysphagia and proximal weakness. Both medications were reintroduced, and the dosage was gradually increased based on his weight. Salbutamol (4 mg per day) was stopped again until June 2017 to observe the effect. However, no differences were reported. Since then, the patient has taken pyridostigmine alone. He has not reported any adverse effects from pyridostigmine. Electrocardiography was routinely performed every 3 months when he was taking salbutamol; the results were all normal.

**Fig. 1:**
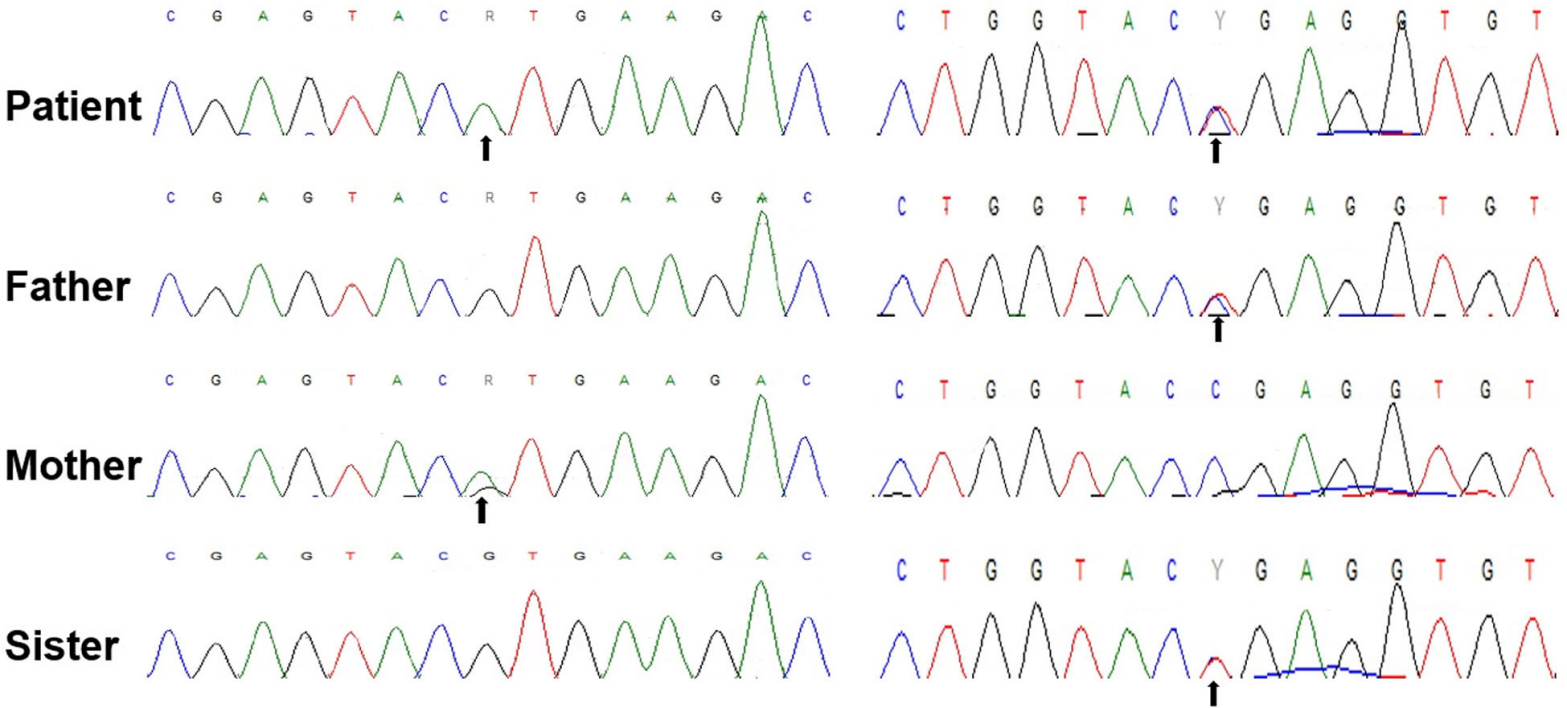
Results of the Sanger sequencing. Electropherograms of the Sanger sequencing show the two heterozygous variants (c.145G > A on the left and c.628C > T on the right) in the patient. The mother was heterozygous for c.145G > A, while the father was heterozygous for c.628C > T. The sister was heterozygous for c.628C > T (p.R210X). The variants are indicated by the black arrows

As the patient had some clinical manifestations of CMS, a neuromuscular genetic panel was first performed. The panel revealed two compound heterozygous missense variants in *PLEC*, which is related to epidermolysis bullosa simplex, although the boy had no history related to that condition. Subsequent genetic testing of the mitochondria from a blood sample was normal, with no evidence of point mutations or deletions/duplications. Then, whole-exome sequencing was performed, leading to the identification of the gene *SLC25A1*. The patient was compound heterozygous for the novel *SLC25A1* variants c.628C > T (p.R210X) and c.145G > A (p.V49M). The father was heterozygous for c.628C > T, while the mother was heterozygous for c.145G > A. These variants were confirmed by Sanger sequencing (Fig. 1). Subsequent Sanger sequencing for 2 *SLC25A1* variants in the sister showed that she was heterozygous for c.628C > T (p.R210X). The patient’s two *SLC25A1* variants have never been reported in the 1000 Genomes Project or the ExAC database. According to the ACMG 2015 guidelines, the c.628C > T (p.R210X) variant, resulting in truncated proteins, was classified as pathogenic (PVS1, PM2, PP4), and the c.145G > A (p.V49M) variant was classified as likely pathogenic (PM2, PM3, PP3, PP4) [8].

Given the combination of his clinical phenotype (fatigable muscular weakness, myasthenic crisis, epilepsy, developmental delay, mild elevation of 2-KG and lactic acid in urine) and the molecular genetic findings, the proband was diagnosed with *SLC25A1*-related disorder. Moreover, we consider the patient’s phenotype to be intermediate between the reported phenotypes of CMS and D/L-2-HGA.

**Fig. 2:**
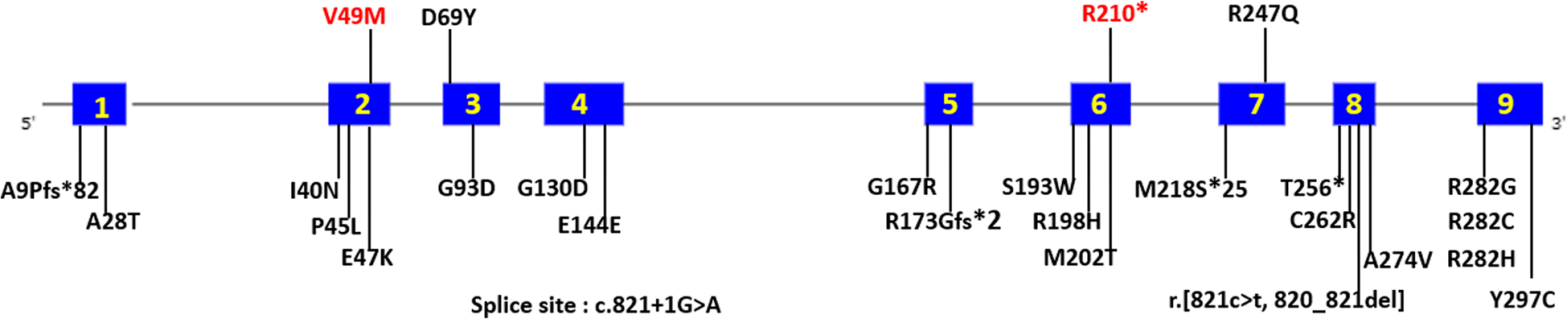
Distribution of 27 novel and previously published *SLC25A1* variants (including our case in red). The upper panel of the schematic shows all pathogenic variants in patients in whom CMS was the main feature, while the mutations in the lower panel correspond to the individuals who had been diagnosed with D/L-2-HGA

Sixteen patients with CMS due to *SLC25A1* have been reported, including our patient (Table 1). All patients had very early onset. The myasthenic features consisted of ocular and proximal limb weakness in all patients, bulbar weakness in 6 patients, and cervical and facial involvement in two patients. Repetitive nerve stimulation (RNS) at 3 Hz elicited a positive response in four patients, and abnormal jitter on nerve block was observed in one patient. Partial responses to acetylcholinesterase (AChE) inhibitors were recorded in three patients, and a partial response to 3,4-diaminopyridine (3,4-DAP) was recorded in one patient. Of three patients, none was responsive to salbutamol. Mild ID was observed in 9 patients. To our knowledge, only 4 out of 27 variants in *SLC25A1* are associated with CMS (Fig. 2).

**Table 1:** Summary of the clinical, neurophysiological, and therapeutic features of CMS cases with *SLC25A1* pathogenic variants

	Reference [5]	Reference [6]	Reference [7]	Our case
**Number of the patients**	2	4	9	1
**Ethnicity**	English	Two Indian; one Greek Pomak; one Pakistani	Arabian	Chinese
**Variants**	R247Q	R247Q	R247Q in 6 pts.; D69Y in 3 pts	R210X; V49M
**Zygosity**	Homozygous	Homozygous	Homozygous	Compound heterozygous
**Age at onset**	Less than 2 years	Infancy	Infancy	9 months
**CMS-associated features**				
**Fatigability**	Yes	Yes	Yes	Yes
**Ocular involvement**	Ptosis and/or diplopia	Ptosis and/or diplopia	Ptosis and/or diplopia	Ptosis and ophthalmoplegia
**Cervical muscle weakness**	Neck flexion weakness in one patient	Neck flexion weakness in one patient	No	No
**Bulbar weakness**	Dysarthria with fatigable speech in one patient	In 2 pts	Stridor in 2 pts.; No stridor in 5 pts.; ND in 2 pts	Yes, stridor and dysarthria
**Limb muscle weakness**	Yes, proximal	Yes, proximal	Yes, proximal	Yes, proximal
**Facial involvement**	In one patient	ND	ND	Yes, chewing weakness
**Myasthenic crises**	No	No	No	Yes, twice
**RNS on 3 Hz stimulation**	Negative in one patient; ND in one patient	Positive in 2 pts.; Negative in 2 pts	Positive in one patient; Negative in 5 pts.; ND in 3 pts	Positive
**Abnormal jitter with block**	Positive in one patient; ND in one patient	Negative in one patient; ND in 3 pts	ND	ND
**Other features**				
**Hypotonia**	No	Hypermobility in two pts	No	Yes
**Epilepsy**	No	No	No	Yes
**Intellectual disability**	Mild	Mild	Mild ID in 2 pts.; No ID in 3 pts.; ND in 2 pts.; NA in 2 pts	Mild
**Urinary organic acids**	Normal	Normal in one patient; ND in 3 pts	Normal in 2 pts.; ND in 7 pts	Mild elevation of 2-oxoglutaric acid and lactate
**Blood lactate levels**	Normal	Elevation after exercise in 2 pts	Normal	Mild elevation
**Muscle biopsy**	Enlarged mitochondria and increased in number on EM in one patient; ND in one patient	Fibre size variation, few central cores and sporadically accumulated mitochondria on EM in one patient; Mildly atrophic fibre, normal COX staining and mild reduction in complex 1 activity in one patient; ND in two pts.	Normal in one patient; Non-specific results in one patient; ND in 7 pts	Atrophy of type IIB muscle fibre with normal COX and SDH staining; No changes in mitochondria on EM
**Responsive to treatment**				
**AChEI**	Partial response in one patient; No response in one patient	Partial response in one patient; No response in 3 pts	No response in 2 pts.; ND in 7 pts	Yes, Partial
**3,4-DAP**	Good response in one patient; ND in one patient	ND	ND	ND
**Salbutamol**	ND	No response in 2 pts.; ND in 2 pts	ND	No

*Abbreviations*: *ND* not determined, *Pts* patients, *RNS* repetitive nerve stimulation, *ID* intellectual disability, *EM* electron microscopy, *AChEI* acetylcholinesterase inhibitor, *3,4-DAP* 3,4-diaminopyridine, *COX* cytochrome c oxidase, *SDH* succinate dehydrogenase, *NA* not applicable

## Discussion and conclusions

Here, we report that a typical CMS patient with some features of D/L-2-HGA harboured novel pathogenic variants in *SLC25A1*.

*SLC25A1*, located on chromosome 22q11, encodes the mitochondrial citrate carrier SLC25A1 (CIC). This protein belongs to the SLC25 family of mitochondrial carriers (MCs), which are mainly localized in the inner mitochondrial membrane and are responsible for the trafficking of a variety of metabolites [9, 10]. The locus of this gene (22q11.2) has also attracted some attention, as it is either translocated or amplified in some tumours and deleted in DiGeorge syndrome and possibly schizophrenia [11, 12]. Since 2013, *SLC25A1* has been reported to be linked to human diseases. Pathogenic variants in *SLC25A1* cause the combined D/L-2-HGA [2–4] and CMS phenotype (MIM) [5, 6] with a recessive mode of inheritance.

The phenotype of *SLC25A1*-related diseases should be a consecutive spectrum consisting of CMS, D/L-2-HGA and the intermediate type (with combined features of CMS and D/L-2-HGA). The diagnosis of congenital myasthenia is based on the presence of early-onset fatigable weakness, impaired NMJ transmission, absence of autoantibodies and response to treatment [13, 14]. Our patient has generalized fluctuating weakness, a positive response to RNS, and negative AChR antibodies, and he manifests some response to pyridostigmine. The myasthenic crises in our patient suggest that clinicians should take respiratory distress or bulbar exacerbation as a warning sign. In addition to the typical manifestation of CMS, the patient had some other features related to the other polarity of *SLC25A1* deficiency, D/L-2-HGA. The boy had occasional epilepsy, hypotonia, and abnormal metabolic signs in the urine and blood.

The origin of the elevated D and L enantiomers of 2-HG due to *SLC25A1* is still unclear, but one proposed explanation is the accumulation of citrate and other tricarboxylic acid (TCA) cycle intermediates in the mitochondria, including 2-KG, which is converted to D- and L-2-HG [15]. There was a slight elevation of 2-KG and lactate levels in our patient’s urine. In addition, two out of four patients in a previous study had increased lactate levels after exercise [6]. This result suggested that CMS due to *SLC25A1* might involve a defect that mildly reduces mitochondrial function. Further studies are required to explain whether there is any correlation between NMJ defects and abnormal metabolite levels.

To our knowledge, only four mutations in *SLC25A1* have been detected in CMS patients to date. Meanwhile, more than 20 pathogenic variants are reportedly related to D/L-2-HGA. What is the relationship between phenotypes and *SLC25A1* genotypes? *SLC25A1* knockdown zebrafish showed motility defects and NMJ abnormalities in less severely affected morphants, while additional defects of the brain and heart were observed in more severely affected morphants [5]. In 2018, phenotype–genotype correlation studies of *SLC25A1*, using the residual activity of missense variants compared to wild-type transfectants, showed that some patients with D/L-2-HGA harboured variants that caused a mild reduction in protein activity [16]. Thus, protein activity does not provide the full story.

In conclusion, we expand the range of known phenotypes and genotypes associated with *SLC25A1*-related disorders. The phenotypes of *SLC25A1*-related diseases seem to be a consecutive spectrum consisting of CMS, D/L-2-HGA and the intermediate type (combining features of CMS and D/L-2-HGA). Future functional studies of *SLC25A1* are needed to delineate the phenotypic differences.

## Supplementary information

**Additional file 1.** Muscle biopsy. A–D Skeletal muscle sections from the patient were stained with COX (A), HE(B), MGT2 (C) and SDH (D) to visualize mitochondria. E-F Electron microscopy findings.

## Data Availability

The data used to support the findings of this study are available from the corresponding author upon request, without breaching participant confidentiality.

## Abbreviations

D/L-2-HGA: D-2 and L-2-hydroxyglutaric aciduria
CMS: Congenital myasthenic syndrome
ID: Intellectual disability
2-KG: 2-ketoglutarate
WES: Whole-exome sequencing
NMJ: Neuromuscular junction
ACMG: American College of Medical Genetics
EEG: Electroencephalogram
AChR: Acetylcholine receptor
COX: Cytochrome c oxidase
SDH: Succinate dehydrogenase
CMAP: Compound muscle action potentials
RNS: Repetitive nerve stimulation
AChE: Acetylcholinesterase
3,4-DAP: 3,4-diaminopyridine
TCA: Tricarboxylic acid
CIC: Citrate carrier SLC25A1
MC: Mitochondrial carrier

## References

[1] Kaplan RS, Mayor JA, Wood DO (1993). The mitochondrial tricarboxylate transport protein. cDNA cloning, primary structure, and comparison with other mitochondrial transport proteins. J Biol Chem.

[2] Nota B, Struys EA, Pop A (2013). Deficiency in SLC25A1, encoding the mitochondrial citrate carrier, causes combined D-2- and L-2- hydroxyglutaric aciduria. Am J Hum Genet.

[3] Cohen I, Staretz-Chacham O, Wormser O (2018). A novel homozygous SLC25A1 mutation with impaired mitochondrial complex V: possible phenotypic expansion. Am J Med Genet A.

[4] Smith A, McBride S, Marcadier JL (2016). Severe neonatal presentation of mitochondrial citrate carrier (SLC25A1) deficiency. JIMD Rep.

[5] Chaouch A, Porcelli V, Cox D (2014). Mutations in the mitochondrial citrate carrier SLC25A1 are associated with impaired neuromuscular transmission. J Neuromuscul Dis.

[6] Balaraju S, Töpf A, McMacken G (2020). Congenital myasthenic syndrome with mild intellectual disability caused by a recurrent SLC25A1 variant. Eur J Hum Genet.

[7] Al-Futaisi A, Ahmad F, Al-Kasbi G (2020). Missense mutations in SLC25A1 are associated with congenital myasthenic syndrome type 23. Clin Genet.

[8] Richards S, Aziz N, Bale S (2015). Standards and guidelines for the interpretation of sequence variants: a joint consensus recommendation of the American College of Medical Genetics and Genomics and the Association for Molecular Pathology. Genet Med.

[9] Palmieri F (2013). The mitochondrial transporter family SLC25: identification, properties and physiopathology. Mol Asp Med.

[10] Palmieri F, Monné M (2016). Discoveries, metabolic roles and diseases of mitochondrial carriers: a review. Biochim Biophys Acta.

[11] Maynard TM, Meechan DW, Dudevoir ML (2008). Mitochondrial localization and function of a subset of 22q11 deletion syndrome candidate genes. Mol Cell Neurosci.

[12] Williams NM, Spurlock G, Norton N (2002). Mutation screening and LD mapping in the VCFS deleted region of chromosome 22q11 in schizophrenia using a novel DNA pooling approach. Mol Psychiatry.

[13] Hantai D, Nicole S, Eymard B (2013). Congenital myasthenic syndromes: an update. Curr Opin Neurol.

[14] Beeson D, Hantai D, Lochmuller H (2005). 126th international workshop: congenital myasthenic syndromes, 24-26 September 2004, Naarden, the Netherlands. Neuromuscul Disord.

[15] Kranendijk M, Struys EA, Salomons GS (2012). Progress in understanding 2-hydroxyglutaric acidurias. J Inherit Metab Dis.

[16] Pop A, Williams M, Struys EA (2018). An overview of combined D-2- and L-2-hydroxyglutaric aciduria: functional analysis of CIC variants. J Inherit Metab Dis.

