# Supplementary material for: A case report of an intermediate phenotype between congenital myasthenic syndrome and D-2- and L-2-hydroxyglutaric aciduria due to novel SLC25A1 variants
Source: BMC Neurol. 2020 Jul 13;20:278. doi: 10.1186/s12883-020-01854-6 (PMC7359281; doi:10.1186/s12883-020-01854-6)
Supplement: Supplementary file 1 — Additional file 1. Muscle biopsy. A–D Skeletal muscle sections from the patient were stained with COX (A), HE(B), MGT2 (C) and SDH (D) to visualize mitochondria. E-F Electron microscopy findings. [file 12883_2020_1854_MOESM1_ESM.docx]

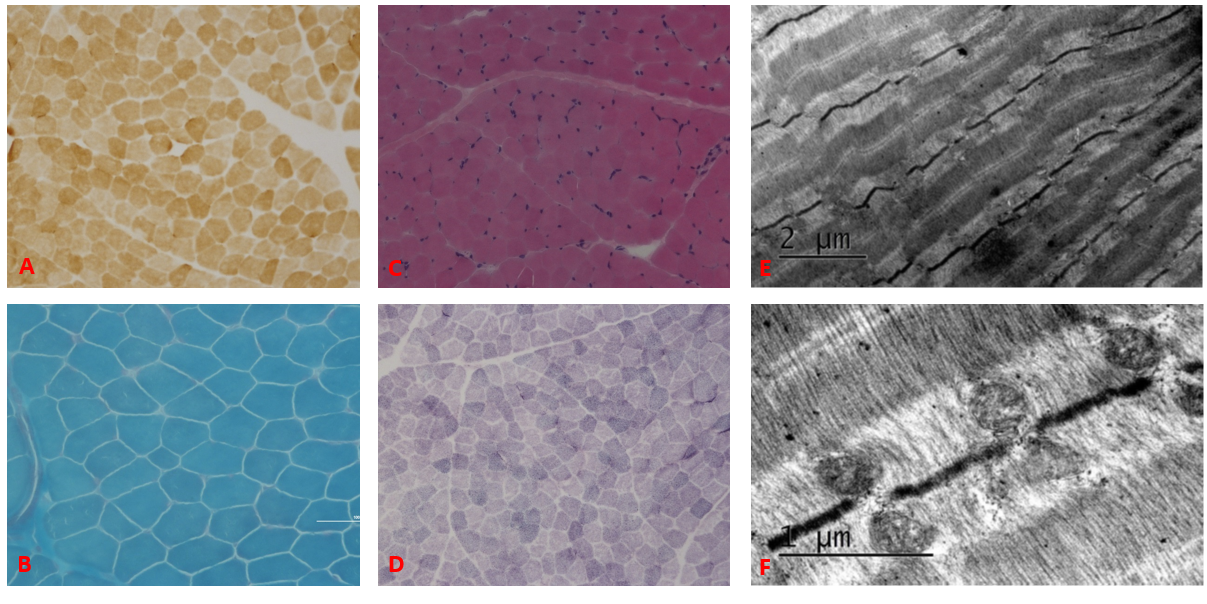


Supplementary data 1 Muscle biopsy. A–D Skeletal muscle sections from the patient were stained with COX (A), HE(B), MGT2 (C) and SDH (D) to visualize mitochondria. E-F Electron microscopy findings.
